# Efficacy and safety of ROH-101 (0.15% ganciclovir gel) for cytomegalovirus corneal endotheliitis: an open-label, uncontrolled, phase 3 study in Japan

**DOI:** 10.1007/s10384-025-01168-5

**Published:** 2025-04-09

**Authors:** Noriko Koizumi, Dai Miyazaki, Seiichiro Sugita, Chie Sotozono, Tsutomu Inatomi, Hiroshi Goto, Atsushi Shiraishi, Shuichiro Eguchi, Shin-ichiro Ito, Yuichi Hori, Eiichi Uchio, Takeshi Soma, Takeo Fukuchi, Ken Hayashi, Yusuke Takeuchi, Yoshitsugu Inoue

**Affiliations:** 1 Department of Biomedical Engineering, Faculty of Life and Medical Sciences, Doshisha University, Kyotanabe, Kyoto 610-0321 Japan; 2 Division of Ophthalmology and Visual Science, Faculty of Medicine, Tottori University, Tottori, Japan; 3 Sugita Eye Hospital, Nagoya, Aichi Japan; 4 Department of Ophthalmology, Kyoto Prefectural University of Medicine, Kyoto, Japan; 5 Department of Ophthalmology, National Center for Geriatrics and Gerontology, Obu, Aichi Japan; 6 Department of Ophthalmology, Tokyo Medical University, Tokyo, Japan; 7 Department of Ophthalmology, Ehime University Graduate School of Medicine, Toon, Ehime Japan; 8 Eguchi Eye Hospital, Hakodate, Hokkaido Japan; 9 Department of Ophthalmology, Kobe City Eye Hospital, Kobe, Hyogo Japan; 10 Department of Ophthalmology, Toho University Graduate School of Medicine, Tokyo, Japan; 11 Department of Ophthalmology, Fukuoka University School of Medicine, Fukuoka, Japan; 12 Department of Ophthalmology, Osaka University Graduate School of Medicine, Osaka, Japan; 13 Department of Ophthalmology and Visual Science, Graduate School of Medical and Dental Sciences, Niigata University, Niigata, Japan; 14 Hayashi Eye Hospital, Fukuoka, Japan; 15 Rohto Pharmaceutical Co., Ltd., Osaka, Japan

**Keywords:** CMV DNA copy number, Cytomegalovirus corneal endotheliitis, Efficacy and safety, ROH-101, 0.15% ganciclovir gel

## Abstract

**Purpose:**

Cytomegalovirus (CMV) corneal endotheliitis often causes severe visual impairment owing to irreversible corneal endothelial dysfunction. Given the side effects of systemic antiviral therapy, development of an approved topical antiviral agent for CMV corneal endotheliitis is desirable. This study evaluated the efficacy and safety of topical 0.15% GCV gel, ROH-101, in the treatment of CMV corneal endotheliitis in Japanese patients.

**Study design:**

Open-label, multicenter, uncontrolled, phase 3 study (jRCT2051210064).

**Methods:**

The study was conducted from August 2021 to December 2022, with a 2-week run-in period with 0.1% fluorometholone eye drops alone, a 12-week treatment period with additional ROH-101, and a 24-week post-treatment observation period after discontinuation of ROH-101. The primary endpoint was the proportion of patients achieving a CMV DNA copy number in the aqueous humor of less than 10^3^ copies/mL at week 12. The clinical findings and safety were assessed over the treatment and post-treatment observation periods.

**Results:**

Twelve eyes of 12 patients with PCR-proven CMV corneal endotheliitis were enrolled. Treatment was discontinued in 1 eye owing to an adverse event. The other 11 eyes completed 12 weeks of treatment with 63.6% achieving the primary endpoint. The clinical findings, such as corneal edema, coin-shaped lesions, and anterior chamber inflammation, improved in all 11 eyes and did not worsen in 8 eyes that completed the post-treatment observation period. Endothelial cell density was well maintained, and none of the 11 eyes showed corneal endothelial dysfunction. Mild adverse drug reactions were reported in 3 eyes (8.3%).

**Conclusion:**

ROH-101 was a safe and efficacious treatment in Japanese patients diagnosed with CMV corneal endotheliitis.

**Supplementary Information:**

The online version contains supplementary material available at 10.1007/s10384-025-01168-5.

## Introduction

Corneal endotheliitis causes specific inflammation of the corneal endothelium, often leading to severe visual impairment owing to irreversible corneal endothelial dysfunction [1]. Corneal endotheliitis has been conventionally considered to develop owing to the reactivation of a herpes simplex virus (HSV) [2, 3] or varicella zoster virus (VZV) infection [4]. In 2006, we reported the first case of cytomegalovirus (CMV)-associated corneal endotheliitis in a healthy individual, confirmed by detection of CMV DNA in the aqueous humor by means of polymerase chain reaction (PCR), who showed a positive response to systemic ganciclovir [5]. Since then, CMV has been increasingly recognized as a significant cause of intractable corneal endotheliitis in immunocompetent patients [6–10].

CMV endotheliitis is typically characterized by corneal edema, coin-shaped lesions, anterior chamber (AC) inflammation, and intraocular pressure (IOP) elevation. It has also been noted in individuals who have undergone corneal transplantation and is considered a potential cause of early post-transplant graft failure [8, 11–13]. Early diagnosis and treatment are essential to prevent irreversible corneal endothelial dysfunction.

Antiviral agents such as ganciclovir (GCV) and valganciclovir hydrochloride have shown effectiveness in treating CMV corneal endotheliitis, highlighting their therapeutic value [14–16]. GCV, which inhibits viral DNA synthesis and replication, is effective against HSV, VZV, and CMV [17]. However, systemic GCV use can lead to severe side effects such as bone marrow suppression and renal failure. Therefore, topical GCV is considered a preferable initial treatment approach. Unfortunately, no GCV eye drops are formally approved for CMV corneal endotheliitis, leading to hospital-dispensing of GCV solutions or off-label use of 0.15% GCV gel, neither covered by health insurance in Japan or other countries. This highlights the urgent need for formally approved topical GCV eye drops and standardized treatments for CMV corneal endotheliitis.

A 0.15% GCV ophthalmic gel is approved for acute herpetic keratitis in over 30 countries [18]. Although it is not approved for CMV corneal endotheliitis, clinical research supports its use for the management of CMV corneal endotheliitis and anterior uveitis, thus avoiding systemic GCV side effects [19, 20]. The therapeutic value of 0.15% GCV gel for CMV corneal endotheliitis is increasingly recognized, but safety data remain scarce [21]. Here, we present findings on the efficacy and safety of ROH-101, a topical 0.15% GCV gel, in Japanese patients with CMV corneal endotheliitis.

## Patients and methods

### Study design

**Fig. 1:**
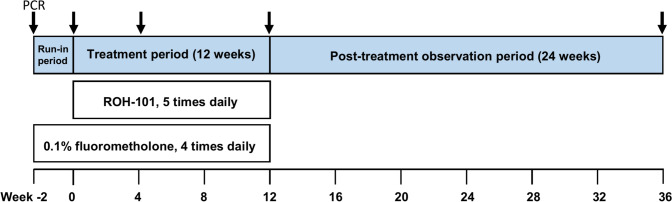
Study design. This study consisted of a run-in period for 2 weeks (from week − 2 to week 0), a treatment period for 12 weeks (from week 0 to week 12), and a post-treatment observation period for 24 weeks (from week 12 to week 36). The patients received 0.1% fluorometholone eye drops alone in the run-in period and in combination with ROH-101 in the treatment period. ROH-101 was discontinued in the post-treatment observation period, but corticosteroid eye drops were continued as needed

This study was an open-label, uncontrolled, phase 3, multicenter clinical trial designed to assess the efficacy and safety of ROH-101 in Japanese patients with CMV corneal endotheliitis. It was conducted across 13 sites from August 2021 to December 2022 with a trial structure that included a 2-week run-in period (from week − 2 to week 0), a 12-week treatment period (from week 0 to week 12), and a 24-week post-treatment observational period (from week 12 to week 36), as depicted in Fig. 1.

The trial was strictly conducted according to the Declaration of Helsinki principles, Good Clinical Practice guidelines, and the Act on Securing Quality, Efficacy, and Safety of Products Including Pharmaceuticals and Medical Devices. Before the study, and as necessary during its course, the ethics committees reviewed and approved the study protocol and informed consent form. Participation in the study was contingent upon each patient providing written informed consent to ensure that ethical standards were maintained throughout the trial. The study was registered with the Japan Registry of Clinical Trials under the identifier jRCT2051210064.

### Study participants

The patients in this study were diagnosed with CMV corneal endotheliitis on the basis of the clinical manifestations and through PCR testing for CMV of the aqueous humor according to the diagnostic criteria for CMV endotheliitis [15]. The included patients also had CMV DNA copy numbers of at least 10^3^ copies/mL, had an endothelial cell density (ECD) of 500 cells/mm^2^ or more, were aged 20 years or older at the time of consent, were capable of having aqueous humor sampled by AC puncture, and had provided written informed consent to participate in the clinical trial. Patients who had received anti-CMV agents within 4 weeks before the start of the observation were excluded from this study. The inclusion and exclusion criteria are outlined in Table 1.

**Table 1:** Inclusion and exclusion criteria

Inclusion criteria	Exclusion criteria
(1) CMV corneal endotheliitis diagnosed on the basis of the clinical manifestations of 1) or 2) 1) Coin-shaped lesions (white keratic precipitate (KP)-like lesions arranged in a small circular or ring pattern) or linear KPs similar to the rejection lines 2) Corneal edema with KPs and 2 or more of the following 3 criteria: decreased endothelial cell density (ECD), recurrent or chronic iridocyclitis, and a history of current or past ocular hypertension	(1) PCR test results positive for HSV or VZV DNA in the aqueous humor
(2) PCR test results positive for CMV DNA in the aqueous humor with CMV DNA copy numbers of at least 10^3^ copies/mL	(2) Corneal endotheliitis caused by other than CMV
(3) ≥ 500 cells/mm^2^ of ECD	(3) Edema in the entire cornea
(4) Aged ≥20 years at the time of consent	(4) Symptoms other than CMV corneal endotheliitis preclude ECD measurement of the central corneal area (eg, corneal scarring)
(5) Capable of sampling aqueous humor by anterior chamber puncture	(5) Anti-CMV agents (GCV, valganciclovir, letermovir, foscarnet sodium hydrate) were administered by any route of administration within 4 weeks before the start of observation
(6) Written informed consent for participation in this clinical trial obtained	(6) Patients with an allergy to GCV
	(7) Patients with CMV retinitis
	(8) Received any treatment agents for other clinical trials within 12 weeks before the start of observation
	(9) Pregnant or attempting to become pregnant or breastfeeding
	(10) Unable to use appropriate contraception (oral contraceptives, condoms, etc) during the run-in period and treatment period (from week − 2 to week 12)
	(11) Judged unsuitable for this clinical trial by the investigators

*CMV* cytomegalovirus, *GCV* ganciclovir, *HSV* herpes simplex virus, *VZV* varicella zoster virus

### Intervention

The patients received 0.1% fluorometholone eye drops alone in the 2-week run-in period and were then started on ROH-101 (a 0.15% ganciclovir gel) for the 12-week treatment period. During that time, 0.1% fluorometholone was continued in combination with ROH-101. The ROH-101 was administered as a single drop 5 times daily (once every 2–3 hours as the standard use), and the 0.1% fluorometholone eye drops were administered at 1–2 drops 4 times daily (once every 3 hours as the standard use). The ROH-101 was discontinued in the post-treatment observation period, whereas the corticosteroid eye drops were continued as needed.

During the study period, the concomitant use of topical and systemic antiviral agents, such as GCV, valganciclovir, letermovir, foscarnet sodium hydrate, acyclovir, valacyclovir, famciclovir, vidarabine, and amenamevir, was prohibited to avoid interference with the evaluation of the efficacy and safety of ROH-101. Similarly, the use of immunosuppressants and corticosteroids, with the exception of the 0.1% fluorometholone eye drops, was restricted up to week 12 or until treatment discontinuation. These concomitant prohibitions were established to ensure an accurate evaluation of ROH-101 without any confounding effects from other systemic or local therapies.

### Endpoints and outcome measures

The primary endpoint of this study was the proportion of patients achieving a CMV DNA copy number in the aqueous humor of less than 10^3^ copies/mL at week 12. Secondary endpoints included changes in CMV DNA copy numbers in the anterior humor, clinical findings observed by slit-lamp examinations (corneal edema, coin-shaped lesions, linear keratic precipitates [KPs] similar to rejection lines, other forms of KPs, and AC inflammation), and assessments of the best-corrected visual acuity (BCVA), IOP, ECD, and central corneal thickness (CCT) throughout the study period.

The participants were scheduled for a total of 11 visits, ranging from week − 2 to week 36. Comprehensive ophthalmologic evaluations, including slit-lamp examination, BCVA, IOP, ECD, and CCT measurements, were conducted at each visit and upon discontinuation of the study. PCR testing for CMV DNA in the aqueous humor was performed at critical time points: at baseline (week − 2), then at weeks 0, 4, 12, 36, and upon study discontinuation, as illustrated in Fig. 1.

Safety assessments included monitoring for adverse events (AEs) and evaluation of vital signs (heart rate and blood pressure) and laboratory test results. AEs were documented at every visit throughout the treatment and post-treatment observation periods. The AEs were categorized according to the primary system organ classes and preferred terms, using the Japanese version of the Medical Dictionary for Regulatory Activities (*MedDRA*, v 25.1).

### Quantitative analysis of CMV DNA by means of real-time PCR

Approximately 100 µL of aqueous humor was collected from the AC of each eye via AC puncture. The CMV DNA copy numbers were quantified by LSI Medience Corporation (Tokyo, Japan) by use of real-time PCR. The methodologies employed were consistent with those developed by Miyazaki and colleagues at Tottori University [22–24]. The process commenced with DNA extraction and was followed by amplification targeting the glycoprotein B gene of CMV by use of the LightCycler 480 II system (Roche). Fifty microliters of aqueous humor was extracted for DNA by use of the QIAamp DNA Mini Kit (Qiagen). The extracted DNA was eluted with 50 µL of water. Then, 8 µL of the extracted DNA was added to each PCR tube. The copy number was determined on the basis of the established dilutions of cloned glycoprotein B DNA. A threshold of less than 1.0 copies/µL (10^3^ copies/mL) was set to designate a sample as CMV DNA negative on the basis of the results of validation testing by LSI Medience Corporation. The standard curve for the validation testing is shown in Table S1.

### Statistical analysis

Statistical analyses for this study were conducted using SAS software, version 9.4 (SAS Institute). The required sample size was estimated by setting of parameters with a control rate of 23%, an expected success rate of 67%, a first-type error of 0.05 (2-sided), and a second-type error of 0.20 while using a binomial test for the null hypothesis. This calculation resulted in a target sample size of 12 patients.

The study included patients with CMV corneal endotheliitis who were treated with ROH-101 and assessed for efficacy at least once in the full analysis set (FAS), per-protocol set (PPS), and safety analysis set (SAF).

The primary endpoint was calculated as the rate of patients showing a decrease in CMV DNA copy numbers in the anterior humor to less than 10^3^ copies/mL at the end of treatment (week 12). A binomial test was then performed with a threshold of 23% and a type 1 error (2-tailed) of 0.05 as the null hypothesis. We calculated the 95% CIs using Sterne’s methods.

The secondary endpoints were calculated as the frequency distribution (number of patients, percentage) of improvements in corneal edema, coin-shaped lesions, linear KPs, other forms of KPs, and AC inflammation at each assessment time point from baseline (week 0). The summary statistics (means and standard deviations [SDs]) were also calculated for the CMV DNA copy number changes in the aqueous humor and for IOP, ECD, CCT, and BCVA. The samples below the cut-off (10^3^ copies/mL) for the CMV DNA copy number were treated as 0 copies/mL (logarithmically treated as 1).

## Results

### Patient disposition

**Fig. 2:**
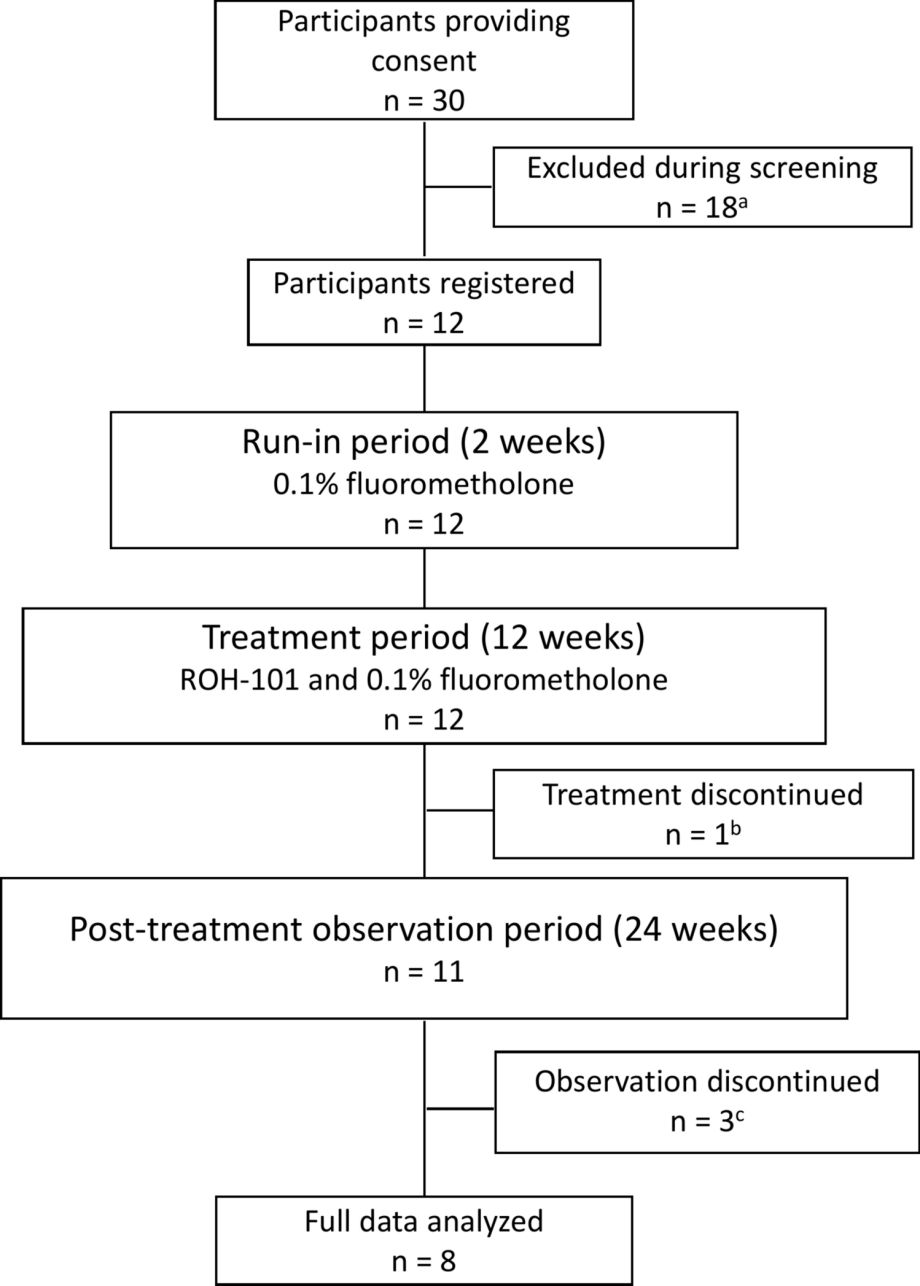
Diagram of patient disposition. **a** Eighteen patients were excluded from the study for the following reasons: CMV DNA copy numbers less than 10^3^ copies/mL (n = 16), consent withdrawn (n = 1), and not suitable for the trial owing to the need for systemic steroid use for other disease (n = 1). **b** One patient discontinued the study treatment owing to adverse events. **c** Two patients discontinued the post-treatment observation owing to worsening of symptoms of CMV corneal endotheliitis, and 1 patient discontinued treatment observation for personal reasons

Informed consent was obtained from 30 patients with clinically suspected CMV corneal endotheliitis. Of these, 12 eyes of 12 patients who met all the inclusion criteria and did not fall into any of the exclusion criteria were enrolled in the ROH-101 clinical trial. All 12 patients were included in the FAS, the PPS, and the SAF. Eleven patients completed the treatment period; however, 1 patient discontinued owing to an AE (a need for an urgent intervention for malignant glaucoma due to an MedDRA classification unrelated to the ROH-101 administration). Eight patients finished the post-treatment observation period. Three patients did not complete this phase: two owing to worsening symptoms of CMV corneal endotheliitis and one for personal reasons, as illustrated in Fig. 2.

### Patient demographics and clinical characteristics at registration

The mean age of the 12 patients enrolled in this study was 72.2 ± 9.2 years. The cohort was predominantly male (91.7%; 11 patients). All the cases involved unilateral corneal endotheliitis. The detailed patient demographics, ocular complications, and past ocular history are summarized in Table S2. Notably, all the patients (100%) had a history of current or past ocular hypertension. At the time of registration, corticosteroid eye drops were administered to 9 of the 12 eyes (75.0%). The duration of CMV corneal endotheliitis after diagnosis was unknown in three of the 12 cases owing to long-term histories, while the mean of the remaining 9 cases was 1.8 ± 4.4 years. Three of these 9 patients were first diagnosed with corneal endotheliitis at the time of enrollment in this study. Anterior segment inflammation, including anterior uveitis and corneal endotheliitis, caused by CMV is a chronic and slow-progressing disease; therefore, collecting accurate information is difficult regarding when corneal endotheliitis develops during the course of CMV anterior uveitis.

The clinical manifestations and CMV DNA copy numbers in the aqueous humor at the time of registration are presented in Table 2. Coin-shaped lesions and linear KPs were observed in 9 (75.0%) and 5 eyes (41.7%), respectively. Other forms of KPs were present in 10 eyes (83.3%). Corneal edema and AC inflammation were noted in 7 (58.3%) and 8 (66.7%) eyes, respectively. The BCVA ranged from 0.05 to 1.5 in decimal fractions. The IOP varied from 5.0 to 31.0 mm Hg, with a mean of 19.7 ± 6.8 mm Hg. Glaucoma medications were used in 10 eyes (83.3%), and high IOP (≥ 20 mm Hg) was observed in 7 eyes (58.3%) despite medication. The mean ECD and CCT were recorded as 1189 ± 607 cells/mm^2^ and 550 ± 60 μm, respectively. The CMV DNA copy number in the aqueous humor ranged from 1.3 × 10^3^ to 9.6 × 10^5^. These clinical findings and the CMV DNA copy numbers met the study’s inclusion criteria.

**Table 2:** Clinical manifestations and CMV copy numbers at registration (week − 2)

Case no.	Coin-shaped lesions	Linear KPs	Other forms of KPs	Corneal edema	AC inflammation	BCVA	IOP*	ECD	CCT	CMV DNA copy number**
1	+	–	+	+	–	0.05	22.7 (4+Tb)	569	562	9.6 × 10^5^
2	+	–	+	–	+	0.3	15.0 (0)	1889	555	2.1 × 10^4^
3	+	–	+	+	–	0.7	20.0 (5+Tb)	702	592	6.6 × 10^4^
4	+	+	–	+	+	0.5	13.0 (2)	686	475	1.4 × 10^4^
5	–	+	+	+	+	NA	26.0 (2)	NA	627	6.1 × 10^3^
6	+	–	+	–	+	1	23.7 (1+Tb)	2636	556	7.1 × 10^3^
7	–	+	–	–	+	0.6	31.0 (4)	1980	476	1.3 × 10^3^
8	+	–	+	+	+	0.8	24.7 (1)	801	591	7.4 × 10^4^
9	+	–	+	–	+	1.5	17.0 (0)	1142	556	8.4 × 10^3^
10	–	+	+	–	–	0.6	18.0 (1)	1405	513	2.7 × 10^4^
11	+	–	+	+	+	0.15	5.0 (3)	1436	450	1.1 × 10^5^
12	+	+	+	+	–	0.4	20.3 (5+Tb)	634	602	1.7 × 10^6^

*CMV* cytomegalovirus, *KPs* keratic precipitates, *AC* anterior chamber, *BCVA* best corrected visual acuity (decimal fraction), *IOP* intraocular pressure (mm Hg), *Tb* acetazolamide tablets, *ECD* endothelial cell density (cells/mm^2^), *CCT* central corneal thickness (µm), *NA* not available

*Number of glaucoma eye drops indicated in parentheses

**CMV copy number in aqueous humor (copies/mL)

### Clinical manifestations at baseline, during treatment, and during the post-treatment observation period

**Fig. 3:**
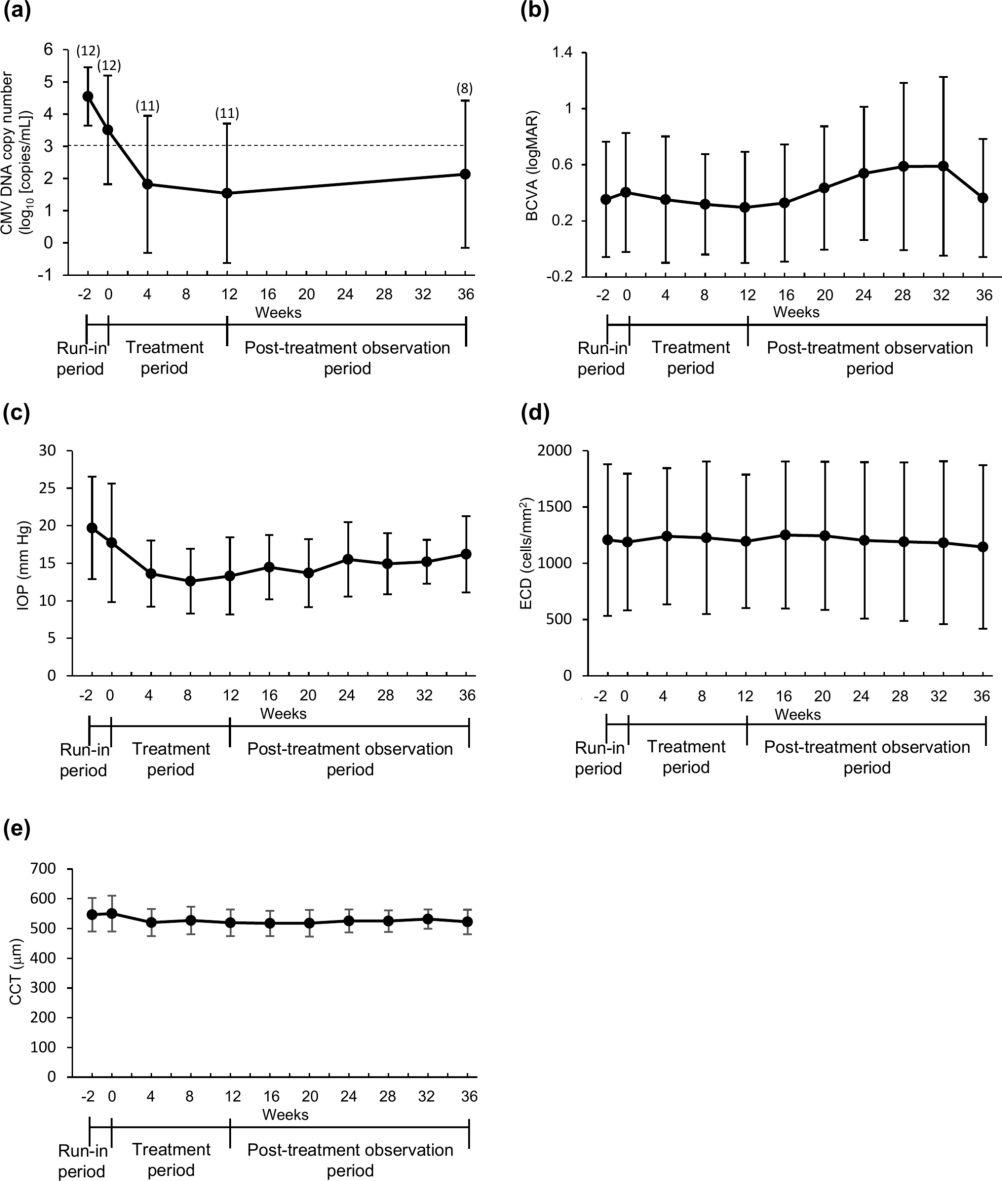
Change in the mean CMV copy number and ophthalmologic examination values. The changes in **a** CMV DNA copy number (log_10_), **b** BCVA (log-MAR), **c** IOP, **d** ECD, and **e** CCT are presented; n = 12 (weeks − 2, 0), 11 (weeks 4, 8, 12, 16), 9 (week 20), 8 (weeks 24, 28, 32, and 36). Data are presented as means ± SDs. The dotted line and (n) in **a** indicate the detection limit and the number of patients. *BCVA* best corrected visual acuity, *CCT* central corneal thickness, *CMV* cytomegalovirus, *ECD* endothelial cell density, *IOP* intraocular pressure

The clinical manifestations and CMV DNA copy numbers at baseline (week 0), during treatment with ROH-101 (weeks 4 and 12), and during the post-treatment period (week 36) are presented in Tables 3 and 4, as well as in Tables S3 and S4. Figure 3 illustrates the means and standard deviations for the CMV DNA copy number, BCVA, IOP, ECD, and CCT at each visit. Case 12 was excluded from the analysis owing to the absence of data from week 4 onward after discontinuation of treatment.

**Table 3:** Clinical manifestations and CMV copy numbers before administration of ROH-101 at week 0

Case no.	Coin-shaped lesions	Linear KPs	Other forms of KPs	Corneal edema	AC inflammation	BCVA	IOP*	ECD	CCT	CMV DNA copy number**
1	+	–	+	+	+	0.05	24.0 (4+Tb)	755	561	1.2 × 10^6^
2	+	–	+	–	–	0.2	34.0 (0)	1849	566	1.9 × 10^4^
3	+	–	+	+	–	0.7	26.0 (5+Tb)	777	583	8.3 × 10^4^
4	+	+	–	+	+	0.3	14.0 (2)	671	509	2.8 × 10^4^
5	–	+	+	+	–	NA	12.7 (1+Tb)	635	592	ND
6	–	–	+	–	+	0.9	17.0 (1+Tb)	2419	552	ND
7	–	+	–	–	+	0.8	10.0 (4+Tb)	2006	463	ND
8	+	–	+	+	–	0.7	15.0 (1)	1191	551	ND
9	+	–	+	+	+	1.5	22.0 (2)	826	626	3.1 × 10^6^
10	–	+	+	+	–	0.6	13.0 (1)	1110	511	3.9 × 10^4^
11	–	–	+	+	+	0.2	5.0 (3)	1401	444	1.3 × 10^5^
12	+	+	+	+	–	0.2	20.0 (5+Tb)	624	643	1.7 × 10^6^

*CMV* cytomegalovirus, *KPs* keratic precipitates, *AC* anterior chamber, *BCVA* best corrected visual acuity (decimal fraction), *IOP* intraocular pressure (mm Hg), *Tb* acetazolamide tablets, *ECD* endothelial cell density (cells/mm^2^), *CCT* central corneal thickness (µm), *ND* not detectable, *NA* not available

*Number of glaucoma eye drops indicated in parentheses

**CMV copy number in aqueous humor (copies/mL)

**Table 4:** Clinical manifestations and CMV copy numbers after 12 weeks of administration of ROH-101 (week 12)

Case no.	Coin-shaped lesions	Linear KPs	Other forms of KPs	Corneal edema	AC inflammation	BCVA	IOP*	ECD	CCT	CMV DNA copy number**
1	Disappeared	–	Improved	Disappeared	Disappeared	0.08	13.3 (4+Tb)	810	514	ND
2	Disappeared	–	Improved	–	–	0.3	25.0 (0)	1773	574	ND
3	Improved	–	Improved	Improved	–	0.8	14.0 (5+Tb)	634	564	ND
4	Improved	Improved	–	Disappeared	Disappeared	NA	11.0 (2)	787	451	ND
5	–	Disappeared	Disappeared	Disappeared	–	NA	9.7 (1)	512	543	ND
6	–	–	Improved	–	Disappeared	1.0	15.3 (0)	2554	538	ND
7	–	Improved	–	–	Improved	1.0	8.0 (4)	1544	453	ND
8	Improved	–	Improved	Disappeared	–	0.8	14.0 (0)	989	543	1.8 × 10^3^
9	Disappeared	–	Improved	Disappeared	Disappeared	1.2	18.0 (0)	1091	554	4.3 × 10^4^
10	–	Improved	Improved	Disappeared	–	0.6	12.0 (1)	1050	518	1.2 × 10^4^
11	–	–	Improved	Improved	Improved	0.2	6.0 (3)	1393	462	8.4 × 10^4^
12	NA	NA	NA	NA	NA	NA	NA	NA	NA	NA

*CMV* cytomegalovirus, *KPs* keratic precipitates, *AC* anterior chamber, *BCVA* best corrected visual acuity (decimal fraction), *IOP* intraocular pressure (mm Hg), *Tb* acetazolamide tablets, *ECD* endothelial cell density (cells/mm^2^), *CCT* central corneal thickness (µm), *ND* not detectable, *NA* not available

*Number of glaucoma eye drops indicated in parentheses

**CMV copy number in aqueous humor (copies/mL)

At baseline (week 0), coin-shaped lesions and linear KPs were detected in 7 (58.3%) and 5 (41.7%) eyes, respectively. Other forms of KPs were present in 10 eyes (83.3%). Nine eyes (75.0%) exhibited corneal edema, and six (50.0%) displayed AC inflammation. Changes in these clinical findings were systematically assessed at weeks 4 and 12 during the ROH-101 treatment period and compared with the baseline measurements.

### Primary endpoint

The proportion of patients whose CMV DNA copy number in the aqueous humor decreased to less than 10^3^ copies/mL was 63.6% (7/11). This was a significant difference because the lower 95% CI exceeded the established threshold of 23% (95% CI = 30.8–89.1; *P* = 0.005). This result showed that ROH-101 effectively reduced CMV DNA levels in the aqueous humor of patients with CMV corneal endotheliitis (Table 5).

**Table 5:** Proportion of patients with efficacy of ROH-101

	N = 11*	95% CI	*P* value
Efficacy**			
Yes	7 (63.6)	30.8–89.1	0.005
No	4 (36.4)	–	

Values are given as n (%)

*One patient was excluded because the data were unavailable owing to discontinuation

**Patients with CMV DNA less than 1.0×10^3^ copies/mL at 12 weeks were judged to be effective

### Secondary endpoints

The mean CMV DNA copy number in the aqueous humor decreased over time during the administration of ROH-101. A slight increase occurred during the post-treatment observation period, as illustrated in Fig. 3a. Improvements in or disappearances of coin-shaped lesions, linear KPs, other forms of KPs, and AC inflammation were observed in 9 of 11 eyes at week 4 (Table S3) and in all the eyes by week 12 (Table 4). These improvements were generally maintained after discontinuation of ROH-101, as shown in Table S4.

Figure 3 summarizes the means and SDs of the CMV DNA copy number changes in the aqueous humor and the IOP, ECD, CCT, and BCVA (logMAR units) measurements. The mean BCVA in logMAR units had improved to 0.30 ± 0.40 by week 12 and had slightly altered, to 0.36 ± 0.42, by week 36 (Fig. 3b). The mean IOP had decreased to 13.3 ± 5.2 mm Hg at week 12 and had stabilized at 16.2 ± 5.1 mmHg at week 36 (Fig. 3c). The mean ECD and CCT values remained stable (Fig. 3d, e).

Although the CMV DNA copy number did not decrease to less than 10^3^ copies/mL in 4 cases after 12 weeks of ROH-101 treatment, all the cases, including those four, exhibited improved clinical outcomes (Table 4). Furthermore, no instances of persistent IOP elevation or irreversible corneal endothelial failure were observed throughout the follow-up period.

### Safety

Detailed descriptions of all the AEs and adverse drug reactions (ADRs) are provided in Table 6. The incidence of AEs was 75.0% (9/12). A serious AE was observed in one (8.3%) of the 12 patients; this was a radius fracture unrelated to the treatment. The most common AEs were eye discharge (2/12 [16.7%]) and arthralgia (2/12 [16.7%]), which were mild and moderate and unrelated to the treatment. Malignant glaucoma was reported as an AE in 1 case, but as noted above, it was unrelated to the treatment. Three ADRs, including eye discharge (1/12 [8.3%]), allergic conjunctivitis (1/12 [8.3%]), and punctate keratitis (1/12 [8.3%]), were mild. The laboratory parameters and vital signs, such as blood pressure and heart rate, remained stable throughout the study period as compared with the baseline, with no clinically significant abnormalities observed from week 2 to week 12.

**Table 6:** Detailed adverse events and adverse drug reactions

Variable	Adverse events	Adverse drug reactions
	(N = 12)	(N = 12)
All events	9 (75.0)	3 (25.0)
Eye disorders	5 (41.7)	3 (25.0)
Eye discharge	2 (16.7)	1 (8.3)
Allergic conjunctivitis	1 (8.3)	1 (8.3)
Conjunctival hemorrhage	1 (8.3)	
Punctate keratitis	1 (8.3)	1 (8.3)
Malignant glaucoma	1 (8.3)	
Gastrointestinal disorders	2 (16.7)	
Abdominal pain upper	1 (8.3)	
Periodontal disease	1 (8.3)	
Hepatobiliary disorders	1 (8.3)	
Cholelithiasis	1 (8.3)	
Infections and infestations	2 (16.7)	
Cystitis	1 (8.3)	
Nasopharyngitis	1 (8.3)	
Esophageal candidiasis	1 (8.3)	
COVID-19	1 (8.3)	
Injury, poisoning, and procedural complications	1 (8.3)	
Radius fracture	1 (8.3)	
Investigations	1 (8.3)	
Blood pressure decreased	1 (8.3)	
Musculoskeletal and connective tissue disorders	2 (16.7)	
Arthralgia	2 (16.7)	
Renal and urinary disorders	1 (8.3)	
Nephrolithiasis	1 (8.3)	
Vascular disorders	1 (8.3)	
Hypertension	1 (8.3)	
Hypotension	1 (8.3)	

MedDRA/J(ver. 25.1)

Values are given as n (%)

## Discussion

The exact pathogenesis of CMV endotheliitis remains elusive; however, it is hypothesized to involve CMV replication within the anterior eye tissues coupled with immune-mediated tissue damage [22–25]. In conditions related to CMV-induced inflammation in the anterior segment, such as corneal endotheliitis and anterior uveitis, the viral load in the AC has been shown to correlate with damage to corneal endothelial cells and inflammation [26, 27]. These observations underscore the critical role that antiviral therapy plays in managing these conditions.

In this clinical trial, the proportion of patients with CMV DNA copy numbers of less than 10^3^ copies/mL in the aqueous humor significantly increased following administration of topical ROH-101 at week 12 (*P* = 0.005). The clinical findings, such as coin-shaped lesions, linear KPs similar to the rejection lines, other forms of KPs, corneal edema, and AC inflammation, also improved from the baseline values or had disappeared at the end of the 12 weeks of treatment. These findings suggest that ROH-101 effectively reduces the CMV viral load in the AC, thereby improving clinical outcomes.

We should note that DNA copy number variation, considered a natural process within the same case, was a challenge in creating the protocol for this study. We actually enrolled 12 cases with CMV DNA copy counts greater than 10^3^ copies at week − 2. However, in four of those cases, the DNA copy number was below the detection limit at week 0 before ROH-101 administration. DNA positivity and the copy number detected in the aqueous humor are known to fluctuate in CMV corneal endotheliitis and anterior uveitis, thereby necessitating multiple repeat anterior chamber aqueous PCR assays to make a definitive diagnosis [23, 28, 29]. Although we cannot rule out the possibility that the corticosteroids administered during the run-in period may have reduced the DNA copy numbers, three of the 4 patients had already received corticosteroids for a long time before entering the study, and their clinical findings were not improved at week 0 before the ROH-101 administration. Therefore, we speculate that the observed reduction in DNA copy number was not due to the corticosteroids but instead reflected fluctuations in the viral load specific to CMV corneal endotheliitis.

A decrease in the DNA copy number and improvement in the clinical findings were also observed in the 7 patients in whom CMV DNA was detected in week 0, indicating that ROH-101 is effective against CMV corneal endotheliitis. Moreover, ROH-101 helped to prevent loss of visual acuity, persistent elevation of IOP, and loss of corneal endothelial cells over the 12-week treatment period without the need for systemic antiviral therapies. The improvements in symptoms achieved with ROH-101 were also sustained for up to 36 weeks during the post-treatment observation period in 8 patients, even without continued use of the medication. It should be noted, however, that two of the patients in which ROH-101 was effective discontinued the study during the postobservation period owing to worsening symptoms of CMV corneal endotheliitis after discontinuation of ROH-101 treatment. Given the propensity for recurrence of CMV corneal endotheliitis, longer follow-up periods after treatment are essential, and resumption of treatment should be considered if symptoms reappear.

In CMV corneal endotheliitis, the inflammation induced by CMV infection can cause acute corneal edema and loss of endothelial cells. Corticosteroid eye drops are frequently used, either before the diagnosis of CMV corneal endotheliitis or alongside antiviral therapy, to reduce inflammation [16, 30, 31]. During this clinical trial, we recognized the common use of corticosteroid eye drops among potential participants and noted their significant impact on treatment outcomes. At registration, corticosteroid eye drops, such as betamethasone or fluorometholone, were being used in 75.0% of the eyes. Consequently, all the study participants underwent a 2-week run-in period with 0.1% fluorometholone eye drops, followed by a 12-week cotreatment period with ROH-101 eye drops. The clinical conditions remained stable during the run-in period with 0.1% fluorometholone eye drops alone. However, after 12 weeks of treatment with ROH-101, the CMV DNA levels were notably reduced, and we saw either improvement or resolution of the clinical endotheliitis signs. In particular, despite the lower GCV concentration with ROH-101 than with the 0.5–2.0% GCV eye drops previously studied [28, 29], ROH-101 was effective in suppressing CMV DNA and improving the clinical symptoms. This efficacy is likely due to the ROH-101 gel formulation, which enhances retention of the drug on the ocular surface and cornea, thereby facilitating sustained therapeutic effects [32–34].

One serious AE, although severe, was unrelated to the ROH-101 treatment, and no deaths occurred in this study. Three patients experienced mild local ocular disturbances, contrasting with the severe systemic side effects of systemic GCV, suggesting ROH-101’s favorable safety profile. Despite the growing awareness of CMV endotheliitis and the increasing number of patients requiring treatment, few reports are available on the safety of GCV eye drops in clinical use. The findings of the present study show that topical antiviral therapy with ROH-101 may be a first-line treatment for CMV corneal endotheliitis in terms of both efficacy and safety.

This study had several notable limitations, including a small patient cohort and the lack of a placebo group owing to ethical concerns over rapid endothelial cell loss and corneal decompensation. These limitations necessitate further research to provide more comprehensive evaluations of the safety and long-term efficacy of ROH-101 across a broader patient population.

## Supplementary Information

Below is the link to the electronic supplementary material.

Supplementary file1 (DOCX 42 KB)
