# Supplementary material for: Efficacy and safety of ROH-101 (0.15% ganciclovir gel) for cytomegalovirus corneal endotheliitis: an open-label, uncontrolled, phase 3 study in Japan
Source: Jpn J Ophthalmol. 2025 Apr 9;69(2):296–307. doi: 10.1007/s10384-025-01168-5 (PMC12003522; doi:10.1007/s10384-025-01168-5)
Supplement: Supplementary file 1 — Supplementary file1 (DOCX 42 KB) [file 10384_2025_1168_MOESM1_ESM.docx]

**Supplementary Table 1** Standard curve of CMV DNA using samples of known concentrations

| Sample no. | DNA concentration, copies/μL | Cp value | Inverse regression value, copies/μL | CV, % | Mean, copies/μL | Relative error, % | Remark |
| --- | --- | --- | --- | --- | --- | --- | --- |
| Standard 1 | 1.0×10^5^ | 16.19 | 9.8×10^4^ | 16.8 | 1.1×10^5^ | 8.0 |  |
|  |  | 16.19 | 9.8×10^4^ |  |  |  |  |
|  |  | 15.76 | 1.3×10^5^ |  |  |  |  |
| Standard 2 | 3.0×10^4^ | 17.97 | 3.1×10^4^ | 7.2 | 3.1×10^4^ | 2.7 |  |
|  |  | 18.05 | 2.9×10^4^ |  |  |  |  |
|  |  | 17.84 | 3.3×10^4^ |  |  |  |  |
| Standard 3 | 1.0×10^4^ | 19.68 | 1.0×10^4^ | 3.3 | 1.0×10^4^ | 0.0 |  |
|  |  | 19.71 | 9.7×10^3^ |  |  |  |  |
|  |  | 19.62 | 1.0×10^4^ |  |  |  |  |
| Standard 4 | 3.0×10^3^ | 21.55 | 2.9×10^3^ | 4.1 | 3.1×10^3^ | 2.0 |  |
|  |  | 21.50 | 3.0×10^3^ |  |  |  |  |
|  |  | 21.42 | 3.2×10^3^ |  |  |  |  |
| Standard 5 | 1.0×10^3^ | 23.11 | 1.1×10^3^ | 3.1 | 1.0×10^3^ | 2.0 |  |
|  |  | 23.18 | 1.0×10^3^ |  |  |  |  |
|  |  | 23.19 | 1.0×10^3^ |  |  |  |  |
| Standard 6 | 3.0×10^2^ | 24.94 | 3.2×10^2^ | 3.6 | 3.1×10^2^ | 3.0 |  |
|  |  | 25.01 | 3.1×10^2^ |  |  |  |  |
|  |  | 25.05 | 3.0×10^2^ |  |  |  |  |
| Standard 7 | 1.0×10^2^ | 26.84 | 9.2×10^1^ | 8.1 | 9.9×10^1^ | -0.9 |  |
|  |  | 26.60 | 1.1×10^2^ |  |  |  |  |
|  |  | 26.77 | 9.7×10^1^ |  |  |  |  |
| Standard 8 | 3.0×10^1^ | 28.57 | 3.0×10^1^ | 8.2 | 2.9×10^1^ | -2.3 |  |
|  |  | 28.50 | 3.1×10^1^ |  |  |  |  |
|  |  | 28.74 | 2.7×10^1^ |  |  |  |  |
| Standard 9 | 1.0×10^1^ | 30.26 | 9.9 | 7.2 | 9.3 | -6.9 |  |
|  |  | 30.33 | 9.5 |  |  |  |  |
|  |  | 30.48 | 8.6 |  |  |  |  |
| Standard 10 | 3.0 | 33.05 | 1.6 | 32.0 | 2.5 | -17.3 |  |
|  |  | 32.02 | 3.1 |  |  |  |  |
|  |  | 32.25 | 2.7 |  |  |  |  |
| Standard 11 | 1.0 | 33.15 | 1.5 | 70.5 | 8.5×10^-1^ | -14.7 |  |
|  |  | 34.21 | 7.5×10^-1^ |  |  |  |  |
|  |  | 35.56 | 3.1×10^-1^ |  |  |  |  |
| Standard 12 | 0.3 | 36.11 | - | - | - | - | E |
|  |  | 35.48 | - |  |  |  |  |
|  |  | ND | - |  |  |  |  |
| Standard 13 | 0.0 | ND | ND | - | - | - | NTC |
|  |  | ND | ND |  |  |  |  |
|  |  | ND | ND |  |  |  |  |

*Cp* crossing point, *CV* coefficient of variation, *ND* not detected, *E* excluded from standard curve, *NTC* negative control

For each standard sample (DNA concentrations ranging from 1.0×10⁵ copies/µL to 0.3 copies/µL), 8 µL was applied to each PCR tube.

**Supplementary Table 2** Demographics, ocular complications, and past ocular history of patients with CMV corneal endotheliitis

| Case no. | Age/sex/eye | Ocular complications | History of ocular disease and surgery* | Steroid eye drops** |
| --- | --- | --- | --- | --- |
| 1 | 62/M/L | Glaucoma, cataract |  | - |
| 2 | 64/M/L | Posner-Schlossmann syndrome, cataract, dry eye |  | 0.1% BM |
| 3 | 85/M/L | Glaucoma, blepharitis, allergic conjunctivitis, dry eye | Cataract | 0.1% BM |
| 4 | 71/M/R | Glaucoma, fungal conjunctivitis, dry eye | Bullous keratopathy | - |
| 5 | 72/M/L | Secondary glaucoma | Cataract, PEA+IOL | 0.1% BM |
| 6 | 67/M/L | Glaucoma | Posner-Schlossmann syndrome, cataract, trabeculotomy, PEA+IOL | - |
| 7 | 86/F/L | Glaucoma, dry eye, PCO |  | 0.1% FM |
| 8 | 72/M/L | Secondary glaucoma, uveitis, cataract, trichiasis | Eyelash removal | 0.1% FM |
| 9 | 58/M/L | Ocular hypertension |  | 0.1% BM |
| 10 | 80/M/L | Glaucoma, dry eye | Anterior uveitis, bullous keratopathy, cataract | 0.1% FM |
| 11 | 67/M/L | Secondary glaucoma, dry eye | Cataract, corneal erosion, keratitis, eyelash removal | 0.1% BM |
| 12 | 82/M/L | Secondary glaucoma, bullous keratopathy | Cataract | 0.1% BM |

*PCO* posterior capsule opacification, *PEA+IOL* phacoemulsification and intraocular lens implantation, *BM* betamethasone, *FM* fluorometholone *History of ocular disease within 5 years and surgery within 8 weeks before enrollment

**Steroid eye drops use at registration (week -2)

**Supplementary Table 3** Clinical manifestations and CMV copy numbers after 4 weeks of administration of ROH-101 (week 4)

| Case no. | Coin-shaped lesions | Linear KPs | Other forms of KPs | Corneal edema | AC inflammation | BCVA | IOP | ECD | CCT | CMV copy number* |
| --- | --- | --- | --- | --- | --- | --- | --- | --- | --- | --- |
| 1 | Disappeared | - | Improved | Improved | Disappeared | 0.08 | 15.7 | 689 | 532 | 3.3×10^3^ |
| 2 | Improved | - | Improved | - | - | 0.2 | 21.0 | 1843 | 555 | ND |
| 3 | Improved | - | Improved | Improved | - | 0.8 | 18.0 | 740 | 560 | 1.2×10^3^ |
| 4 | Improved | Improved | - | Improved | Improved | 0.3 | 11.0 | 734 | 453 | ND |
| 5 | - | Improved | Improved | Improved | - | NA | 10.0 | 515 | 538 | ND |
| 6 | - | - | Improved | - | Disappeared | 1.0 | 14.0 | 2516 | 539 | ND |
| 7 | - | Unchanged | - | - | Unchanged | 1.0 | 11.0 | 1730 | 451 | ND |
| 8 | Improved | - | Improved | Improved | - | 0.9 | 17.0 | 1081 | 555 | ND |
| 9 | Disappeared | - | Improved | Disappeared | Improved | 1.5 | 12.0 | 1048 | 571 | 1.7×10^4^ |
| 10 | - | Improved | Improved | Improved | - | 0.6 | 15.0 | 1382 | 513 | 2.3×10^4^ |
| 11 | - | - | Worsened | Unchanged | Unchanged | 0.1 | 5.0 | 1357 | 456 | 6.4×10^4^ |
| 12 | NA | NA | NA | NA | NA | NA | NA | NA | NA | NA |

*CMV* cytomegalovirus, *KPs* keratic precipitates, *AC* anterior chamber, *BCVA* best corrected visual acuity (decimal fraction), *IOP* intraocular pressure (mm Hg), *ECD* endothelial cell density (cells/mm^2^), *CCT* central corneal thickness (µm), *ND* not detected, *NA* not available

*CMV copy number in aqueous humor (copies/mL)

**Supplementary Table 4** Clinical manifestations and CMV copy numbers after 24 weeks of post-treatment observation (week 36)

| Case No | Coin-shaped lesions | Linear KPs | Other forms of KPs | Corneal edema | AC inflammation | BCVA | IOP* | ECD | CCT | CMV copy number** |
| --- | --- | --- | --- | --- | --- | --- | --- | --- | --- | --- |
| 1 | Disappeared | - | Improved | Disappeared | Disappeared | NA | 15.3 (4) | 783 | 521 | ND |
| 2 | Disappeared | - | Improved | - | - | 0.1 | 10.0 (0) | 1882 | 545 | ND |
| 3 | Improved | - | Improved | Improved | - | 1.2 | 27.0 (5+Tb) | 439 | 583 | 2.6×10^4^ |
| 4 | Improved | Improved | - | Disappeared | Disappeared | 0.4 | 12.0 (2) | 512 | 466 | 1.3×10^4^ |
| 5 | - | Disappeared | Unchanged | Disappeared | - | NA | 15.0 (1) | 555 | 534 | ND |
| 6 | - | - | Improved | - | Disappeared | 1 | 18.7 (0) | 2467 | 549 | ND |
| 7 | NA | NA | NA | NA | NA | NA | NA | NA | NA | NA |
| 8 | NA | NA | NA | NA | NA | NA | NA | NA | NA | NA |
| 9 | NA | NA | NA | NA | NA | NA | NA | NA | NA | NA |
| 10 | - | Improved | Improved | Disappeared | - | 0.7 | 16.0 (1) | 1220 | 517 | 6.0×10^3^ |
| 11 | - | - | Improved | Disappeared | Improved | 0.2 | 15.6 (3) | 1303 | 463 | 5.4×10^4^ |
| 12 | NA | NA | NA | NA | NA | NA | NA | NA | NA | NA |

*CMV* cytomegalovirus, *KPs* keratic precipitates, *AC* anterior chamber, *BCVA* best corrected visual acuity (decimal fraction), *IOP* intraocular pressure (mm Hg), *Tb* acetazolamide tablets, *ECD* endothelial cell density (cells/mm^2^), *CCT* central corneal thickness (µm), *ND,*not detected, *NA* not available

*Number of glaucoma eye drops indicated in parentheses

**CMV copy number in aqueous humor (copies/mL)
